# New horizons in hospital-associated deconditioning: a global condition of body and mind

**DOI:** 10.1093/ageing/afae241

**Published:** 2024-11-04

**Authors:** Carly Welch, Yaohua Chen, Peter Hartley, Corina Naughton, Nicolas Martinez-Velilla, Dan Stein, Roman Romero-Ortuno

**Affiliations:** Department of Twin Research & Genetic Epidemiology, King’s College London, St Thomas’ Campus, 3rd & 4th Floor South Wing Block D, Westminster Bridge Road, London SE1 7EH, UK; Department of Ageing and Health, Guy’s and St Thomas’ NHS Foundation Trust, St Thomas’ Hospital, 9th Floor North Wing, Westminster Bridge Road, London SE1 7EH, UK; Univ Lille, CHU Lille, U1172, Degenerative and Vascular Cognitive Disorders, Department of Geriatrics, Lille, France; Global Brain Health Institute, Trinity College Dublin, Dublin 2, Ireland; Department of Physiotherapy, Cambridge University Hospital NHS Foundation Trust, Cambridge CB2 0QQ, UK; Department of Public Health and Primary Care, University of Cambridge, Cambridge CB2 0SZ, UK; University College Dublin, School of Nursing Midwifery and Health Systems, Health Sciences Centre Belfield, Dublin 4, Ireland; Navarre Health Service (SNS-O), Navarre University Hospital (HUN), Department of Geriatrics, Navarrabiomed, Navarre Public University (UPNA), Navarra Institute for Health Research (IdiSNA), Pamplona, Spain; Department of Twin Research & Genetic Epidemiology, King’s College London, St Thomas’ Campus, 3rd & 4th Floor South Wing Block D, Westminster Bridge Road, London SE1 7EH, UK; Department of Ageing and Health, Guy’s and St Thomas’ NHS Foundation Trust, St Thomas’ Hospital, 9th Floor North Wing, Westminster Bridge Road, London SE1 7EH, UK; Global Brain Health Institute, Trinity College Dublin, Dublin 2, Ireland; Discipline of Medical Gerontology, School of Medicine, Trinity College Dublin, Dublin 2, Ireland

**Keywords:** sarcopenia, delirium, cognitive, muscle, frailty, older people

## Abstract

Hospital-associated deconditioning is a broad term, which refers non-specifically to declines in any function of the body secondary to hospitalisation. Older people, particularly those living with frailty, are known to be at greatest risk. It has historically been most commonly used as a term to describe declines in muscle mass and function (i.e. acute sarcopenia). However, declines in physical function do not occur in isolation, and it is recognised that cognitive deconditioning (defined by delayed mental processing as part of a spectrum with fulminant delirium at one end) is commonly encountered by patients in hospital. Whilst the term ‘deconditioning’ is descriptive, it perhaps leads to under-emphasis on the inherent organ dysfunction that is associated, and also implies some ease of reversibility. Whilst deconditioning may be reversible with early intervention strategies, the long-term effects can be devastating. In this article, we summarise the most recent research on this topic including new promising interventions and describe our recommendations for implementation of tools such as the Frailty Care Bundle.

## Key Points

- Deconditioning is a whole body syndrome affecting cognitive as well as physical function.
- Implementation tools such as the Frailty Care Bundle offer opportunities to improve fundamental care of older people in hospital.
- Interdisciplinary working in both clinical practice and research is vital to accelerate change to reduce adverse outcomes.

## Background

The potential negative consequences of hospitalisation have been known about since the dawn of geriatric medicine as a specialty. Marjory Warren’s multidisciplinary initiative at West Middlesex County Hospital in 1935 was pioneering [1]. She demonstrated that health and wellbeing of older people could be improved through stimulated environments, improved natural lighting, rehabilitation ethos and treatment of potentially reversible pathology [1]. In 1947, Richard Asher published in the *British Medical Journal* on the ‘Dangers of going to bed’, describing multisystem adverse consequences of bedrest [2]. Recent advances in practice of geriatric medicine have increasingly recognised this, with development of new models of care to enable people to receive care and treatment as close to home as possible, including ‘interface geriatrics’ [3], integrated care [4, 5] enhanced community care [6] or ‘Hospital at Home’ services [7, 8]. However, there will always remain a need for some patients to be admitted to hospital for acute treatments, and it is imperative that we ensure our hospitals are designed in a way to minimise burdens of harm.

**Figure 1:**
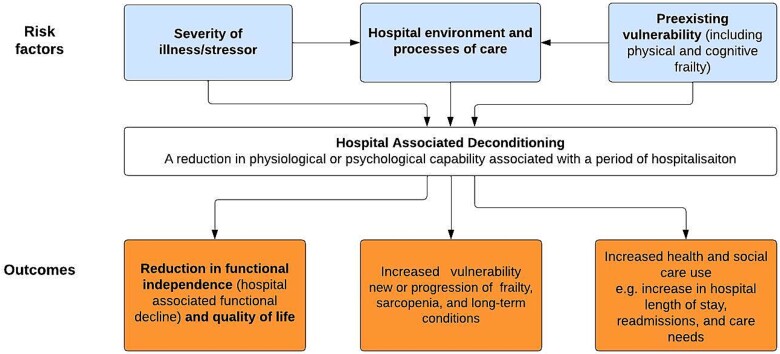
Association of risk factors with hospital-associated deconditioning and resulting outcomes.

Hospital-associated deconditioning is a concept with which both healthcare professionals and patients are familiar [9]. Older people who have spent time in acute hospital often report feeling or more vulnerable afterwards, or that perhaps it took them longer to get back into their normal routines [10]. Whilst these effects are often related to effects of illness itself, there is a need to recognise the role that the acute hospital environment and its processes of care have on compounding these effects [10], and what can be done to mitigate this [11]. Effects are often intensified in vulnerable older people, especially in those already living with frailty and/or cognitive impairments, often manifesting as loss of functional independence (Figure 1). The prevalence of hospital-associated functional decline in a meta-analysis of 15 studies was 30% [12]. Importantly, a previous study in France judged 82% of incident cases to be potentially preventable [13]. These effects may be individually significant and a person who was living with very mild frailty prior to admission may find themselves with moderate frailty upon discharge from hospital [14, 15]. Such patients are likely to have longer lengths of stay in hospital [16] and require increased care needs on discharge [17]. This is clearly important at an individual level for patients, their quality of life and support network [18], as well as being of significance to public health and social care systems, considering inherent costs [7].

From a research perspective, there is tendency for academics to work within silos, either concentrating on individual organ systems, muscle and physical function, or cognitive function. However, nothing in the body works in isolation, and problems in one area will have consequences in another [19]. There is increased understanding of the need for researchers in physical and cognitive functioning to work collaboratively [20]. We held a symposium on ‘Hospital-Associated Deconditioning’ at the European Geriatric Medicine Society congress in London, 2022, bringing together researchers and clinicians across disciplines, and across Europe. In this article, we summarise current understanding of this condition, present our recommendations to clinicians and policymakers on how they can implement changes to improve patient care now, and summarise possible future interventions.

### Physical deconditioning—acute sarcopenia and muscle spectrum disorders

Skeletal muscle is an often-forgotten organ system in many models of disease. Whilst clinicians routinely monitor other organ systems during hospitalisation (e.g. through vital signs or blood tests), monitoring of muscle size, quality or function rarely takes place as part of standard care [21]. Acute sarcopenia is a term that refers to acute muscle insufficiency [22]. It is defined by incident sarcopenia (reduced muscle strength with reduced muscle quantity or quality) within 6 months [23]. It is distinct from chronic age-related sarcopenia, which is defined by progressive declines in muscle mass and function with time. Acute sarcopenia should be considered akin to acute organ dysfunction elsewhere (e.g. acute respiratory failure, acute kidney injury) and prioritised as such [22].

Recent studies demonstrated that one in five older people who do not meet criteria for sarcopenia at baseline meet criteria for sarcopenia on subsequent testing within 1 week of hospitalisation or at discharge [14, 24]. However, trajectories of muscle quantity, quality and function during hospitalisation vary significantly between individuals; individual variations may be more relevant [14, 25]. Acute sarcopenia can be considered part of a spectrum of muscle-wasting disorders occurring with hospitalisation, including intensive care unit acquired weakness, and dynamic changes not fully meeting criteria for acute sarcopenia but which may still be of functional relevance to the individual [22]. Loss of strength in hospital may particularly affect the lower limbs [26]. Loss of strength has been shown to be greatest in patients with the greatest sedentary times, and in patients already living with frailty prior to admission [26]. Additionally, physical activity and nutritional status interact in promotion of muscle loss during hospitalisation [27]. Furthermore, delirium and cognitive spectrum disorders have been shown to be associated with increased risk of declines in muscle quantity and physical function during hospitalisation [28, 29], and prescription of steroids during admission has been associated with increased risk of sarcopenia 1 week after hospitalisation (adjusting for baseline sarcopenia status) [29].

The pathophysiology of acute sarcopenia remains incompletely understood. It is likely that there are multiple underlying processes contributing towards its development. During hospitalisation, acute sarcopenia can arise due to a combination of heightened inflammation, anabolic resistance, reduced protein intake and importantly reduced physical activity—especially bedrest [22]. These factors lead to a cascade of cellular processes that prevent muscle protein synthesis and promote muscle protein breakdown [21]. In patients with delirium and cognitive spectrum disorders, acute sarcopenia may also arise through reduced initiation of motor control [21]. Older adults, and especially those living with frailty, are likely to be more vulnerable due to impaired immune responses (immunosenescence) to promote muscle and tissue repair [30, 31], reduced growth hormone secretion [32], reduced numbers of motor neurones [33] and cellular senescence [34].

### Cognitive deconditioning—cognitive spectrum disorders beyond delirium

Cognitive deconditioning has been historically overlooked in studies of hospital-associated deconditioning. Only 1 out of 22 papers included within a systematic review of hospital-associated deconditioning incorporated cognitive function as a predictor [35]. Cognitive change patterns during hospitalisation have been shown to be heterogenous. In some individuals, cognition declines but subsequently improves, whereas in others cognitive changes may persist at discharge [36]. In addition, undiagnosed cognitive impairment is known to be highly prevalent in hospitalised older people [37]; this is commonly unmasked within unfamiliar and busy hospital environments.

Delirium is an acute severe neuropsychiatric syndrome characterised by changes in consciousness, attention and cognition caused by physical disturbances to the body [38]. Delirium occurs due to a peripheral inflammatory response leading to a secondary central inflammatory response [39]. It is now recognised as the psychiatric manifestation of acute encephalopathy [40]. Delirium is not a benign process—it is associated with increased mortality and increased length of hospital stay in survivors [41]. There is also now strong evidence that delirium leads to worsening cognition over time [42] and that this relates to inflammatory processes [43]. It is known to be more common in people who have dementia and pre-existing cognitive impairment, as well as those who are living with frailty. A previous study showed a stepwise increased risk of delirium with each grade of the Clinical Frailty Scale [44]. Delirium has also been shown to be associated with impaired functional status on discharge. This demonstrates the close bidirectional interplay between delirium and physical function [45].

Delirium is part of a spectrum of cognitive disorders encountered during hospitalisation. The term ‘subsyndromal delirium’ has been denoted to classify psychiatric states that consist of some but not all features of delirium. Subsyndromal delirium has been less fully characterised than delirium, which is complex by its nature in encompassing heterogeneous presentations [46]. However, studies suggest that subsyndromal delirium is associated with increased risk of adverse outcomes [47], albeit to a lesser extent compared to those who meet all criteria for delirium [41]. Indeed, delirium severity has been shown to be predictive of risk of adverse outcomes [48].

Cognitive deconditioning is a broad term without a unified definition, which recognises that even in patients who do not develop delirium [35], hospitalisation and acute illness can lead to impaired cognitive processing that is independent of an inflammatory process. High levels of anxiety and depressive symptoms have been noted in hospitalised older people, with these symptoms commonly persisting after discharge [49]. Psychological distress may in turn lead to maladaptive cognitive responses and reduced engagement with physical activity [36]. This in turn can lead to a vicious cycle since physical activity levels are highly correlated with mood [50] and cognition [51], as well as physical function [52]. These effects may additionally be exacerbated by the use of psychotropic and antipsychotic medications [53].

### Deconditioning as a multisystem disorder

It is clear that deconditioning is a whole-body syndrome [21]. Bedrest promotes constipation even amongst healthy young volunteers [54]. This effect is compounded in older adults [55], where colonic transit times are already delayed [56] and gut microbiome diversity is reduced [57]. Gut microbiome may also be adversely affected by the hospital environment [58] and unvaried nutrition [59]. Constipation in turn can promote urinary retention and the need for urinary catheterisation [60]. However, urinary incontinence may also develop as part of the deconditioning syndrome. Lack of mobilisation and promotion of early toileting can lead to impaired feedback mechanisms of bladder control, particularly where continence pads are used empirically [61]. Urinary incontinence in this context can be challenging to reverse.

Additionally, reduced mobilisation increases the risk of pressure ulcer formation [62]. However, pressure ulcers are just one of the end-points of a spectrum of conditions encompassing skin insufficiency in this context. The effects of this should not be overlooked. Skin dryness caused by ineffective moisturisation alone can lead to impairments in the barrier effect of the skin, increased propensity to infections and impaired wound healing [63]. This can be further compounded by the effects of reduced mobility and inadequate nutrition leading to dependent oedema, particularly in the lower legs, and further risk of skin breakdown [64]. Prolonged bedrest can also lead to orthostatic intolerance, including postural symptoms and orthostatic hypotension during subsequent attempts at mobilisation [65]. These effects can then lead to a vicious cycle of impaired mobilisation due to increased effort to overcome additional weight (from both oedema and adiposity) and postural symptoms.

### Consequences of hospital-associated deconditioning

**Figure 2:**
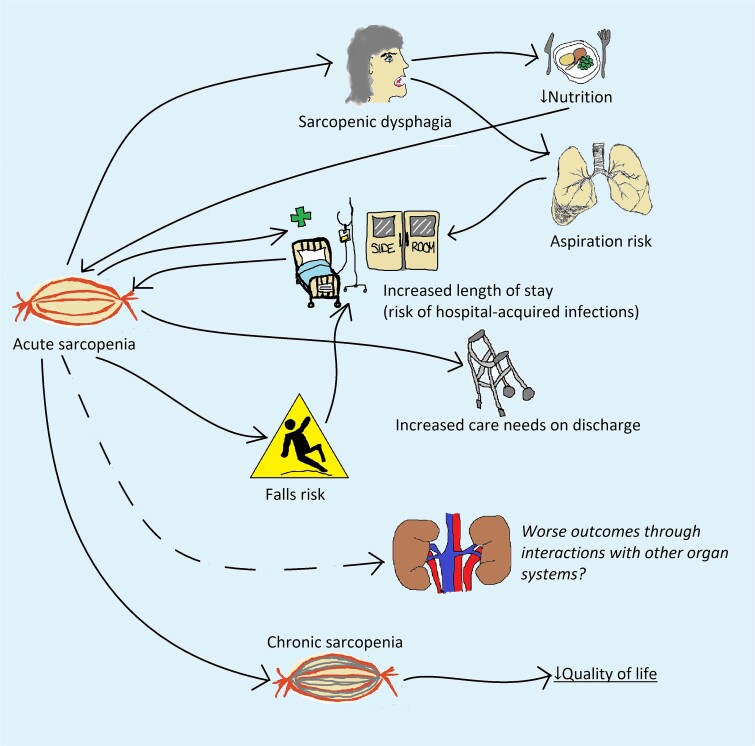
Consequences of acute sarcopenia.

Unfortunately, effects of hospital-associated deconditioning are not always temporary and can be long lasting. In a study of 515 community-living older people hospitalised for acute non-critical medical illness, 53% were unable to walk a quarter of a mile and 61% were unable to drive 6 months after hospitalisation [66]. Figure 2 shows potential effects of acute sarcopenia. Unfortunately, this process can lead to a vicious cycle, whereby reduced muscle strength can lead to increased risk of falls and increased length of hospital stay. Sarcopenia does not just affect appendicular muscles, but can also affect swallowing muscles, leading to sarcopenic dysphagia [67], thereby leading to reduced nutrition and further exacerbation of acute sarcopenia. It is known that people with sarcopenia have increased mortality independently of age, long-term conditions and activities of daily living, i.e. muscle insufficiency independently affects survival [68]. It is increasingly clear that muscles act as secretory organs; if this is affected in acute sarcopenia, it could lead to worse outcomes through impaired communication with other organ systems. Importantly, acute sarcopenia leads to increased risk of long-term development of chronic sarcopenia, leading to reduced quality of life [69].

Delirium is associated with a two-fold increased risk of death during hospital admission [41], but is also associated with an increased risk of new long-term care placement [48], and an eight-fold increased risk of a later life dementia diagnosis [70]. Factors that are most predictive of adverse outcomes in delirium include delirium duration, hypoactive motor subtype, delirium severity, and pre-existing dementia or depression [48]. The Delirium and Cognitive Impact in Dementia study showed that delirium was associated with significant cognitive decline at 1-year follow-up compared to older people without delirium, with patients with more than one episode of delirium or longer duration of delirium experiencing the worse outcomes [42]. Delirium has also been shown to be associated with functional decline at 1 month after hospitalisation, with a gradient of worsening decline seen between subsyndromal delirium and delirium [45].

### What can we do to prevent hospital-associated deconditioning? Introducing the Frailty Care Bundle

The core of interventions to reduce deconditioning are exercise, nutrition and positive cognitive stimulation, all of which are central elements of fundamental care [71, 72]. At ward level, the challenge is consistent implementation against competing clinical and organisational priorities in often understaffed wards and with a workforce that may receive very limited gerontological education in undergraduate or postgraduate curricula [73]. A previous systematic review identified 230 facilitators and 342 barriers to improving physical activity levels during hospitalisation, demonstrating the complexity of this area. Broadly, facilitators include knowledge, awareness, attitudes and interdisciplinary collaboration. Barriers include patient fear, healthcare professional safety concerns and availability of equipment [74]. Drawing on practical learning from implementation of a Frailty Care Bundle in orthopaedic trauma and older adult wards [75], and from the broader evidence [76–81], there are common themes in implementing fundamental care to reduce deconditioning risk. Table 1 provides specific detail as to how and what changes should be implemented.

**Table 1:** Practical guidance on implementation of a Frailty Care Bundle in clinical practice

Leadership	
Ward level	Identify key leaders across disciplines—senior nurse, therapist and consultant with consistent presence on ward Arrange early leadership meeting between disciplinary leaders to clarify vison, agree consistent messages across disciplines and avoid conflicting ideas Senior staff members to champion education of staff within their own discipline with cross-sharing of knowledge from core leadership Role modelling by senior staff e.g. senior medical staff mobilise patients during medical reviews, ward manager assists at mealtimes Organisation of daily interdisciplinary board rounds on ward to include updates on mobilisation status and interprofessional handovers Maintain and monitor safe staffing levels (medical, nursing and therapy), with shared communication of standards across disciplines, using safe staffing frameworks where applicable Involve the wider professional team to include an educational package for volunteers, administrative staff, cleaners, catering staff, porters and students that ensures safety and promotes whole team working
Organisation level	Embedding patient care at the heart of the organisation’s ethos Recognising areas that have prioritised fundamental care Supporting and encouraging the development of ward initiatives, whilst also ensuring wards have the autonomy in oversight Increasing public awareness of deconditioning through posters, written information and web-based information such as video recordings Encouraging a culture that promotes learning from events (e.g. falls) rather than punishment. Learning from events should take a nuanced approach that appreciates complexity of care, whilst striving for improvements
Domains	
Eating	Promoting protected and assisted mealtimes where (unless required for urgent clinical reasons) staff prioritise meal time over competing demands, and enable visitors to support nutrition Ensuring those who require assistance have this available Ensure patients with special nutrition requirements are readily identifiable to nursing and catering staff Ensuring availability of varied meal options including specialised cultural meals where appropriate If patients have low intake, offer alternative high-energy, high-protein snacks All staff on the ward should be provided with education on how to provide safe assistance and understanding of different food consistencies as a core part of the ward educational package Screen for risk of malnutrition on initial admission and weekly thereafter, with early referral to dietetics and/or speech and language therapy when needed Support and encourage patients to sit out at mealtimes and ideally where possible sitting in communal areas with other patients Provide snack and hydration rounds to include protein (e.g. cheese, yogurt, rice pudding, high-protein milkshakes) Tailor food preferences for patients at high nutrition risk (e.g. provide snack boxes for patients with grazing pattern) Time administration of oral nutrition supplements to avoid clash with mealtimes
Mobilisation and activity	Design physical ward environments that promote safe mobilisation and way finding Identification of patient’s pre-admission mobility status including use walking and/or transfer aids used at the point of admission On admission, support patients to continue to mobilise in line with their pre-admission mobility status where possible—physiotherapy assessment may not be required at this stage Early referral to physiotherapy staff to support review within 24 h where there has been a change in mobility compared to pre-admission Do not delay mobilisation while waiting physiotherapy review unless clear indication (e.g. lower limb fracture, bedrest due to medical reason) Consider tools such as ‘traffic light’ systems that will enable all staff members to understand when they can safely assist patients with mobilisation Avoid prolonged periods of bed or chair rest and encourage activity in active patients e.g. seated exercises with dumbbells, pedals, marching and progressive sit-to-stands Promotion of toileting and ensuring careful consideration of all options before providing continence pads to a patient who did not previously have these—treat new incontinence as a serious event requiring assessment and intervention by the multidisciplinary team
Cognition and interaction	All older patients to be screened for delirium on admission using validated tool e.g. 4AT Staff training to enable screening for delirium using tools such as the Single Question in Delirium—‘Is this patient more confused than normal?’ by non-specialised staff (i.e. including students, volunteers, etc.) Clear delirium escalation and management pathway alerting medical staff for immediate review and diagnosis of delirium (or other conditions) following positive screening Design ward areas to ensure natural lighting, with promotion of natural sleep–wake cycles through minimisation of light and noise at night times Ensure clocks and calendars are clearly visible and correctly adjusted Promote activities at the bedspace or in group areas such as day rooms e.g. reading, crosswords, games, drawing Educational programmes to support all staff (in particular to those providing enhanced care) to feel empowered to commence cognitive stimulation activities at any time Encouraging visiting of relatives and advocates

Leadership is the central pillar in enabling ward teams to adapt to new practices. The ward manager and lead clinician with support of the multidisciplinary team are essential in addressing interprofessional silos that may impede effective team working [82]. Stereotypical norms, such as mobilisation being the role of physiotherapists, dieticians looking after nutrition and occupational therapists looking after cognition, can be overcome through a shared team vision of high-quality older adult care [83]. Operationalising the vision requires collective effort through partnership with older people and carers [84], and multidisciplinary quality improvement activity with measurable key performance indicators, capacity for self-monitoring and team feedback which includes bedside nurses, health care assistants and catering staff [85]. Wards can be further supported in the development of initiatives through central organisation leadership and an ethos that values and recognises fundamental patient care.

Communication is the next pillar. Maintaining attention on fundamental care requires efficient and effective communication between the older person, bedside nursing team, the medical team and allied health professional team. Formulating patient-expected goals for fundamental care needs to centrally involve the older person to enable their active participation in their recovery and care planning [84]. In addition, sharing information on patient goals needs to be incorporated into daily nursing team huddles as well as multidisciplinary team meetings to ensure prioritisation against a backdrop of competing demands on staff time and organisational priorities [86]. Central to these processes of course is the patient, with whom communication and shared decision making is of the utmost importance. Healthcare staff should also be educated in how to educate patients themselves, empowering them to engage with early mobilisation and other strategies.

There are a range of inter-related pragmatic strategies to operationalise fundamental care at ward and individual patient level. These include

- Incorporating ‘What Matters’ to the older person as part of individualised care planning [83]
- Assisted and protected mealtimes [87]
- Setting, recording and assisting patients to achieve daily mobility goals [88]
- Designing physical ward environments that promote safe mobilisation and way finding
- Structured positive cognitive stimulation sessions [89]
- Screening for delirium [78]
- Strict monitoring of psychotic drugs [90]

Prioritising and consistently delivering this model of care requires ward staffing levels and skill-mix that match the acuity and dependency of the patient cohort, and use modern safe staffing frameworks [91]. Identification of frailty (e.g. using tools such as the Clinical Frailty Scale) on admission can help in guiding risk of hospital-associated deconditioning and ensuring patients who could most benefit from specialised older persons’ wards are prioritised for admission to these units. Age, Clinical Frailty Scale, dementia and mental health problems at admission have been shown to be associated with increased likelihood of requiring increased care on discharge in hospitalised patients [17].

However, none of the individual elements described above can be considered novel. The multicomponent interdisciplinary principles presented have previously been shown to reduce functional and cognitive decline in models such as the Hospital Elder Life Program [92] and the EAT-WALK-ENGAGE [93] programme. Yet, many older people report feelings of insignificance or powerlessness to influence the care that they receive [94]. Hospital policy often focuses on improvements in efficiency and patient flow between departments. However, multicomponent intervention care models have been shown to be cost-effective in improving patient outcomes through reduced length of hospital stay and fewer adverse events [95].

### Emerging and future interventions

We cannot really talk about innovative models because as far back as the mid-twentieth century, Marjory Warren described the importance of addressing this significant clinical situation through specific exercise programmes, even during hospitalisation [96]. However, for various reasons, this clinical situation remains a common problem that requires an integrative approach [77]. In fact, there are many different consensuses and documents with recommendations for establishing a homogeneous approach [97]. Most approaches, despite improving mobility, are not capable of transforming this improvement into substantial modifications at the level of activities of daily living and quality of life [78, 98]. One possibility to explain these results is through comparisons with how we use pharmaceuticals. Just as when we prescribe a drug, we must individualise and personalise the dose, intervals, frequency, etc., and monitor the response and possible adverse effects, we must individualise and personalise our approaches, and generic consensuses or advice may not be effective.

### Exercise interventions

Innovative alternatives are emerging, such as implementation of specific gyms or rooms with necessary equipment to evaluate and prescribe exercise to hospitalised older adults in a personalised manner. Even in small spaces, small gyms and activities of daily living suites can be integrated into geriatric hospital floors. This approach has demonstrated significant changes not only in physical function and quality of life [99, 100], but also in the transformation of basic activities of daily living, which ultimately can modify life trajectories and capacity [101]. In parallel, this approach has allowed for the modification of cognitive aspects and delirium [102, 103]. Use of pedal exercisers, seated side-tapping and chair-based exercises have shown to be safe and feasible in hospitalised older people [104].

### Cognitive stimulation and motivation

Individual responses to exercise programmes can be heterogeneous [105]. Another emerging model is use of technology and games [106]. A three-armed non-randomised controlled trial was conducted at a tertiary public hospital in Navarra, Spain. Participants were allocated to one of three groups: a simple gamification group consisting of 21 participants, a technology-based gamification group with 23 participants or a control group comprising 26 participants. Results indicated that the technology-based gamification intervention programme may offer significant benefits in physical and muscle function compared to usual care, potentially reversing the functional decline commonly associated with acute hospitalisation in older adults [106]. In addition, the modification of environments can be proposed to induce promotion of walking. Social and cultural aspects may require us to personalise strategies, but different models have been established. Activities and interaction between patients can be encouraged through supported use of day rooms including for meals, and activities facilitators [107].

### Nutrition

The importance of adequate protein intake is well recognised for promotion of muscle synthesis and thus prevention of acute sarcopenia [108]. Nutritional interventions have also been used for prevention of delirium as part of multicomponent interventions [109]. However, evidence from clinical trials remains uncertain [104, 109]. This is likely related to the heterogeneity of deconditioning syndromes and a need to take an individualised approach. Branched-chain amino acids may be appropriate interventions to ameliorate muscle [110] and cognitive spectrum disorders [111]. However, much in the same way as exercise interventions, effectiveness is likely to require an individualised approach in terms of dose and timing. Beyond protein supplementation, other specific nutritional interventions that have been trialled in hospitalised populations of older people include β-hydroxy-β-methylbutyrate, oral nutritional supplementation, eicosapentaenoic acid, citrulline and nitrate-rich beetroot juice [104].

### Pharmaceutical

At present, there is no one medication patients can take that can prevent or treat any form of hospital-associated deconditioning. Medications that have been trialled in the context of acute declines in muscle quantity or function include testosterone [112] and anabolic steroids [113]. Medications that have been trialled for delirium include antipsychotics [114], steroids [115] and anti-inflammatories [116]. Whilst there have been some drug trials suggesting potential efficacy of some of these medications, drug trials have historically analysed clinical outcomes through comparisons within overall study populations. Research to identify the underlying mechanistic pathways involved within deconditioning syndromes may enable more targeted interventions towards preventing long-term negative consequences. Deconditioning may be associated with accelerated ageing [117], and measuring dynamic changes in the underlying hallmarks of ageing may enable the early delivery of medications to improve long-term quality of life (e.g. drugs targeted towards senescence) [118]. There are likely to be many different pathways involved and in future a stratified medicine approach may enable more holistic individualised treatment.

### Other interventions

Neuromuscular electrical stimulation (NMES) is a proposed intervention to prevent acute sarcopenia. It works by directly stimulating the muscle to contract and generate growth. Evidence for NMES to prevent declines in muscle dysfunction is currently moderate [119–121]; however, it could not be used as a strategy for deconditioning as a whole, as it is unlikely to offer multisystem benefits.

### Implementation science and novel identification tools

Despite increasing availability of tools such as handheld dynamometers, there remain multiple obstacles to implementation of functional monitoring during hospitalisation [122]. The Hierarchical Assessment of Balance and Mobility was initially proposed in 1995 and has been validated as a reliable tool offering visualisation of functional trajectories in a similar fashion to traditional vital sign observation charts [123]. However, this has not been widely implemented in clinical practice [124]. An implementation science approach is needed to bridge the gap between academia and clinical practice. An emerging approach within health informatics research is the use of live electronic patient record data to make predictions. These predictions aim to assist with clinical decision making, often using machine learning methods. Models have been applied to predictions of clinical deterioration [125], predictors of admission from the emergency department [126] and hospital length of stay [127]. Applying this approach to identification of patients at risk of or at early stages of hospital-associated deconditioning (e.g. through language models developed using free text) could assist clinicians in decision making to prevent adverse outcomes. Another emerging tool is the use of remote monitoring devices such as accelerometers to identify changes in physical activity levels during hospitalisation [27]. In the future, step count could be incorporated within electronic healthcare data and predictive models could be developed to identify when activity levels are lower than would be expected based on factors such as frailty status. Research incorporating these novel models should incorporate economic assessments to ensure cost-effectiveness.

## Conclusions

Hospital-associated deconditioning affects the entire body. Preventative strategies should therefore utilise a multidimensional approach, such as the Frailty Care Bundle. These approaches require a multiprofessional approach whereby all healthcare professionals recognise the role they have to contribute. Simple measures that can be implemented within clinical practice immediately include promotion of early mobilisation and nutrition, considerations of the hospital environment such as availability of natural lighting, delirium screening and prevention, improving communication between staff, patients and relatives, and promoting activities such as creative engagement. Future research is needed across the breadth of the translational pathway. Discovery science and early translational research should focus on deciphering underlying fundamental mechanisms towards development of targeted intervention strategies. Applied health research should focus on testing of complex interventions in diverse populations including exercise, nutritional strategies, and even novel or repurposed pharmaceutical agents. Lastly, implementation science approaches are needed to embed routine monitoring of muscle strength, physical function and cognitive function into clinical practice, alongside currently best evidenced practices of early mobilisation and holistic care plans.

## References

[1] Ritch A . History of geriatric medicine: from Hippocrates to Marjory Warren. J R Coll Physicians Edinb. 2012;42:368–74. 23240126 10.4997/JRCPE.2012.417

[2] Asher RAJ . Dangers of going to bed. Br Med J. 1947;2:967–8. 18897489 10.1136/bmj.2.4536.967 PMC2056244

[3] Oo MT, D'Costa D. Interface geriatrics: modernising conventional geriatric medical care. Clin Med (Lond). 2012;12:99–100. 22372242 10.7861/clinmedicine.12-1-99 PMC4953441

[4] Kjelsnes A, Feiring E. Models of integrated care for older people with frailty: a horizon scanning review. BMJ Open. 2022;12:e060142. 10.1136/bmjopen-2021-060142 PMC8996021 35396317

[5] Bajeux E, Corvol A, Somme D. Integrated care for older people in France in 2020: findings, challenges, and prospects. Int J Integr Care. 2021;21:16. 10.5334/ijic.5643 PMC8588900 34824565

[6] Health Service Executive . Enhanced Community Care 2023. Ireland: Health Service Executive. [online] Available from: https://www.hse.ie/eng/services/list/2/primarycare/enhanced-community-care/.

[7] Singh S, Gray A, Shepperd S et al. Is comprehensive geriatric assessment hospital at home a cost-effective alternative to hospital admission for older people. Age Ageing. 2021;51:afab220. 10.1093/ageing/afab220 PMC8753046 34969074

[8] Levine D, Ouchi K, Blanchfield B et al. Hospital-level care at home for acutely ill adults. Ann Intern Med. 2020;172:77–85. 31842232 10.7326/M19-0600

[9] Guilcher SJT, Everall AC, Cadel L et al. A qualitative study exploring the lived experiences of deconditioning in hospital in Ontario. Canada BMC Geriatrics. 2021;21:169. 33750320 10.1186/s12877-021-02111-2 PMC7941932

[10] Krumholz HM . Post-hospital syndrome—an acquired, transient condition of generalized risk. N Engl J Med. 2013;368:100–2. 23301730 10.1056/NEJMp1212324 PMC3688067

[11] Smith TO, Sreekanta A, Walkeden S et al. Interventions for reducing hospital-associated deconditioning: a systematic review and meta-analysis. Arch Gerontol Geriatr. 2020;90:104176. 32652367 10.1016/j.archger.2020.104176

[12] Loyd C, Markland AD, Zhang Y et al. Prevalence of hospital-associated disability in older adults: a meta-analysis. J Am Med Dir Assoc. 2020;21:455–61.e5. 31734122 10.1016/j.jamda.2019.09.015 PMC7469431

[13] Sourdet S, Lafont C, Rolland Y et al. Preventable iatrogenic disability in elderly patients during hospitalization. J Am Med Dir Assoc. 2015;16:674–81. 25922117 10.1016/j.jamda.2015.03.011

[14] Welch C, Greig C, Majid Z et al. Induced frailty and acute sarcopenia are overlapping consequences of hospitalisation in older adults. J Frailty Sarcopenia Falls. 2022;07:103–16. 10.22540/JFSF-07-103 PMC9433945 36119557

[15] Amblàs-Novellas J, Torné A, Oller R et al. Transitions between degrees of multidimensional frailty among older people admitted to intermediate care: a multicentre prospective study. BMC Geriatr. 2022;22:722. 36050635 10.1186/s12877-022-03378-9 PMC9438217

[16] Juma S, Taabazuing MM, Montero-Odasso M. Clinical frailty scale in an acute medicine unit: a simple tool that predicts length of stay. Can Geriatr J. 2016;19:34–9. 27403211 10.5770/cgj.19.196 PMC4922366

[17] Geriatric Medicine Research Collaborative, Covid Collaborative . Age and frailty are independently associated with increased COVID-19 mortality and increased care needs in survivors: results of an international multi-centre study. Age Ageing. 2021;50:617–30. 33543243 10.1093/ageing/afab026 PMC7929433

[18] Crocker TF, Brown L, Clegg A et al. Quality of life is substantially worse for community-dwelling older people living with frailty: systematic review and meta-analysis. Qual Life Res. 2019;28:2041–56. 30875008 10.1007/s11136-019-02149-1 PMC6620381

[19] Romero-Ortuño R, Martínez-Velilla N, Sutton R et al. Network physiology in aging and frailty: the grand challenge of physiological reserve in older adults. Front Netw Physiol. 2021;1:712430. 10.3389/fnetp.2021.712430 PMC10012993 36925570

[20] Beeri MS, Leugrans SE, Delbono O et al. Sarcopenia is associated with incident Alzheimer's dementia, mild cognitive impairment, and cognitive decline. J Am Geriatr Soc. 2021;69:1826–35. 33954985 10.1111/jgs.17206 PMC8286176

[21] Welch C. Acute sarcopenia: definition and actual issues. In: Veronese N, Beaudart C, Sabico S, editors. Sarcopenia: Research and Clinical Implications. Cham: Springer International Publishing; 2021. p. 133–43, 10.1007/978-3-030-80038-3_10.

[22] Welch C, Hassan-Smith ZK, Greig CA et al. Acute sarcopenia secondary to hospitalisation - an emerging condition affecting older adults. Aging Dis. 2018;9:151–64. 29392090 10.14336/AD.2017.0315 PMC5772853

[23] Cruz-Jentoft AJ, Bahat G, Bauer J et al. Sarcopenia: revised European consensus on definition and diagnosis. Age Ageing. 2019;48:16–31. 30312372 10.1093/ageing/afy169 PMC6322506

[24] Martone AM, Bianchi L, Abete P et al. The incidence of sarcopenia among hospitalized older patients: results from the Glisten study. J Cachexia Sarcopenia Muscle. 2017;8:907–14. 28913934 10.1002/jcsm.12224 PMC5700449

[25] Welch C, Greig C, Lewis D et al. Trajectories of muscle quantity, quality and function measurements in hospitalized older adults. Geriatr Gerontol Int. 2022;22:311–8. 35246911 10.1111/ggi.14366 PMC9313889

[26] Hartley P, Romero-Ortuno R, Wellwood I et al. Changes in muscle strength and physical function in older patients during and after hospitalisation: a prospective repeated-measures cohort study. Age Ageing. 2020;50:153–60. 10.1093/ageing/afaa103 32902637

[27] Welch C, Greig C, Lewis D et al. Baseline nutritional status and in-hospital step count are associated with muscle quantity, quality, and function: results of an exploratory study. J Nutr Gerontol Geriatr. 2023;42:110–26. 37787986 10.1080/21551197.2023.2259335

[28] Hartley P, Gibbins N, Saunders A et al. The association between cognitive impairment and functional outcome in hospitalised older patients: a systematic review and meta-analysis. Age Ageing. 2017;46:559–67. 28119313 10.1093/ageing/afx007

[29] Welch C, Bravo L, Gkoutos G et al. Establishing predictors of acute sarcopenia: a proof-of-concept study utilising network analysis. Aging Dis. 2024;16: [ePub ahead of print]. 10.14336/AD.2024.0167. PMC12221406 39012665

[30] Wilson D, Drew W, Jasper A et al. Frailty is associated with neutrophil dysfunction which is correctable with phosphoinositol-3-kinase inhibitors. J Gerontol A Biol Sci Med Sci. 2020;75:2320–25. 10.1093/gerona/glaa216 PMC7662170 32877922

[31] Wilson D, Jackson T, Sapey E et al. Frailty and sarcopenia: the potential role of an aged immune system. Ageing Res Rev. 2017;36:1–10. 28223244 10.1016/j.arr.2017.01.006

[32] Junnila RK, List EO, Berryman DE et al. The GH/IGF-1 axis in ageing and longevity. Nat Rev Endocrinol. 2013;9:366–76. 23591370 10.1038/nrendo.2013.67 PMC4074016

[33] Gonzalez-Freire M, de Cabo R, Studenski SA et al. The neuromuscular junction: aging at the crossroad between nerves and muscle. Front Aging Neurosci. 2014;6:208. 25157231 10.3389/fnagi.2014.00208 PMC4127816

[34] van Deursen JM . The role of senescent cells in ageing. Nature. 2014;509:439–46. 24848057 10.1038/nature13193 PMC4214092

[35] Chen Y, Almirall-Sánchez A, Mockler D et al. Hospital-associated deconditioning: not only physical, but also cognitive. Int J Geriatr Psychiatry. 2022;37:1–13. 10.1002/gps.5687 PMC9303382 35142397

[36] Chen CC, Chiu MJ, Chen SP et al. Patterns of cognitive change in elderly patients during and 6 months after hospitalisation: a prospective cohort study. Int J Nurs Stud. 2011;48:338–46. 20403601 10.1016/j.ijnurstu.2010.03.011

[37] Jackson TA, MacLullich AMJ, Gladman JRF et al. Undiagnosed long-term cognitive impairment in acutely hospitalised older medical patients with delirium: a prospective cohort study. Age Ageing. 2016;45:493–9. 27076525 10.1093/ageing/afw064

[38] American Psychiatric Association . *Diagnostic and Statistical Manual of Mental Disorders*. 5th edition, Arlington, Virginia, USA: American Psychiatric Publishing, Inc., 2013.

[39] Wilson JE, Mart MF, Cunningham C et al. Delirium. Nat Rev Dis Primers. 2020;6:90. 33184265 10.1038/s41572-020-00223-4 PMC9012267

[40] Slooter AJC, Otte WM, Devlin JW et al. Updated nomenclature of delirium and acute encephalopathy: statement of ten societies. Intensive Care Med. 2020;46:1020–2. 32055887 10.1007/s00134-019-05907-4 PMC7210231

[41] Geriatric Medicine Research Collaborative . Delirium is prevalent in older hospital inpatients and associated with adverse outcomes: results of a prospective multi-centre study on world delirium awareness day. BMC Med. 2019;17:229. 31837711 10.1186/s12916-019-1458-7 PMC6911703

[42] Richardson SJ, Davis DHJ, Stephan BCM et al. Recurrent delirium over 12 months predicts dementia: results of the Delirium and Cognitive Impact in Dementia (DECIDE) study. Age Ageing. 2021;50:914–20. 33320945 10.1093/ageing/afaa244 PMC8099011

[43] Krogseth M, Davis D, Jackson TA et al. Delirium, neurofilament light chain, and progressive cognitive impairment: analysis of a prospective Norwegian population-based cohort. Lancet Healthy Longev. 2023;4:e399–408. 37459878 10.1016/S2666-7568(23)00098-3

[44] Geriatric Medicine Research Collaborative . Increasing frailty is associated with higher prevalence and reduced recognition of delirium in older hospitalised inpatients: results of a multi-centre study. Eur Geriatr Med. 2023;14:325–32. 36696045 10.1007/s41999-022-00737-y PMC10113325

[45] Martínez-Velilla N, Bouzon CA, Contin KC et al. Different functional outcomes in patients with delirium and subsyndromal delirium one month after hospital discharge. Dement Geriatr Cogn Disord. 2012;34:332–6. 23208559 10.1159/000345609

[46] Sepulveda E, Leonard M, Franco JG et al. Subsyndromal delirium compared with delirium, dementia, and subjects without delirium or dementia in elderly general hospital admissions and nursing home residents. Alzheimers Dement. 2017;7:1–10. 10.1016/j.dadm.2016.11.002 PMC5233793 28116342

[47] Gao Y, Gao R, Yang R et al. Prevalence, risk factors, and outcomes of subsyndromal delirium in older adults in hospital or long-term care settings: a systematic review and meta-analysis. Geriatr Nurs. 2022;45:9–17. 35286871 10.1016/j.gerinurse.2022.02.021

[48] Jackson TA, Wilson D, Richardson S et al. Predicting outcome in older hospital patients with delirium: a systematic literature review. Int J Geriatr Psychiatry. 2016;31:392–9. 26302258 10.1002/gps.4344

[49] Walker FB, Novack DH, Kaiser DL et al. Anxiety and depression among medical and surgical patients nearing hospital discharge. J Gen Intern Med. 1987;2:99–101. 3559782 10.1007/BF02596305

[50] Choi KW, Chen CY, Stein MB et al. Assessment of bidirectional relationships between physical activity and depression among adults: a 2-sample Mendelian randomization study. JAMA Psychiatry. 2019;76:399–408. 30673066 10.1001/jamapsychiatry.2018.4175 PMC6450288

[51] Kumar M, Srivastava S, Muhammad T. Relationship between physical activity and cognitive functioning among older Indian adults. Sci Rep. 2022;12:2725. 35177736 10.1038/s41598-022-06725-3 PMC8854730

[52] Nascimento MM, Gouveia ÉR, Marques A et al. The role of physical function in the association between physical activity and gait speed in older adults: a mediation analysis. Int J Environ Res Public Health. 2022;19:12581. 10.3390/ijerph191912581 PMC9564593 36231881

[53] Chandramouleeshwaran S, Khan WU, Inglis F et al. Impact of psychotropic medications on cognition among older adults: a systematic review. Int Psychogeriatr. 2023; [published online] 1–18. 10.1017/S1041610223000844. 37860872

[54] Iovino P, Chiarioni G, Bilancio G et al. New onset of constipation during long-term physical inactivity: a proof-of-concept study on the immobility-induced bowel changes. PloS One. 2013;8:e72608. 23977327 10.1371/journal.pone.0072608 PMC3748072

[55] Salari N, Ghasemianrad M, Ammari-Allahyari M et al. Global prevalence of constipation in older adults: a systematic review and meta-analysis. Wien Klin Wochenschr. 2023;135:389–98. 36826591 10.1007/s00508-023-02156-w

[56] Madsen JL, Graff J. Effects of ageing on gastrointestinal motor function. Age Ageing. 2004;33:154–9. 14960431 10.1093/ageing/afh040

[57] Badal VD, Vaccariello ED, Murray ER et al. The gut microbiome, aging, and longevity: a systematic review. Nutrients. 2020;12:3759. 10.3390/nu12123759 PMC7762384 33297486

[58] Aardema H, Lisotto P, Kurilshikov A et al. Marked changes in gut microbiota in cardio-surgical intensive care patients: a longitudinal cohort study. Front Cell Infect Microbiol. 2019;9:467. 32010644 10.3389/fcimb.2019.00467 PMC6974539

[59] David LA, Maurice CF, Carmody RN et al. Diet rapidly and reproducibly alters the human gut microbiome. Nature. 2014;505:559–63. 24336217 10.1038/nature12820 PMC3957428

[60] Rego R, Barroso D, Freire E. Prevalence of constipation on an internal medicine ward. Aging Med. 2023;6:98–9. 10.1002/agm2.12244 PMC10000263 36911089

[61] Palmer MH, Baumgarten M, Langenberg P et al. Risk factors for hospital-acquired incontinence in elderly female hip fracture patients. J Gerontol A. 2002;57:M672–7. 10.1093/gerona/57.10.m672 12242323

[62] Wann-Hansson C, Hagell P, Willman A. Risk factors and prevention among patients with hospital-acquired and pre-existing pressure ulcers in an acute care hospital. J Clin Nurs. 2008;17:1718–27. 18578778 10.1111/j.1365-2702.2008.02286.x

[63] Jiang Q, Wang Y, Liu Y et al. Prevalence and associated factors of dry skin among older inpatients in hospitals and nursing homes: a multicenter cross-sectional study. Int J Nurs Stud. 2022;135:104358. 36152467 10.1016/j.ijnurstu.2022.104358

[64] Besharat S, Grol-Prokopczyk H, Gao S et al. Peripheral edema: a common and persistent health problem for older Americans. PloS One. 2021;16:e0260742. 34914717 10.1371/journal.pone.0260742 PMC8675752

[65] Wahba A, Shibao CA, Muldowney JAS et al. Management of orthostatic hypotension in the hospitalized patient: a narrative review. Am J Med. 2022;135:24–31. 34416163 10.1016/j.amjmed.2021.07.030 PMC8688312

[66] Dharmarajan K, Han L, Gahbauer EA et al. Disability and recovery after hospitalization for medical illness among community-living older persons: a prospective cohort study. J Am Geriatr Soc. 2020;68:486–95. 32083319 10.1111/jgs.16350 PMC7735402

[67] Fujishima I, Fujiu-Kurachi M, Arai H et al. Sarcopenia and dysphagia: position paper by four professional organizations. Geriatr Gerontol Int. 2019;19:91–7. 30628181 10.1111/ggi.13591

[68] Beaudart C, Zaaria M, Pasleau F et al. Health outcomes of sarcopenia: a systematic review and meta-analysis. PloS One. 2017;12:e0169548. 28095426 10.1371/journal.pone.0169548 PMC5240970

[69] Beaudart C, Reginster JY, Petermans J et al. Quality of life and physical components linked to sarcopenia: the SarcoPhAge study. Exp Gerontol. 2015;69:103–10. 25979160 10.1016/j.exger.2015.05.003

[70] Davis DH, Muniz Terrera G, Keage H et al. Delirium is a strong risk factor for dementia in the oldest-old: a population-based cohort study. Brain. 2012;135:2809–16. 22879644 10.1093/brain/aws190 PMC3437024

[71] Zisberg A, Shadmi E, Gur-Yaish N et al. Hospital-associated functional decline: the role of hospitalization processes beyond individual risk factors. J Am Geriatr Soc. 2015;63:55–62. 25597557 10.1111/jgs.13193

[72] Kitson A, Carr D, Conroy T et al. Speaking up for fundamental care: the ILC Aalborg statement. BMJ Open. 2019;9:e033077. 10.1136/bmjopen-2019-033077 PMC6924742 31822543

[73] Naughton C, Hayes N, Ezhova I et al. Evaluation of the feasibility of an education-career pathway in healthcare for older people (ECHO) for early career nurses. Int J Older People Nurs. 2023;18:e12526. 36658469 10.1111/opn.12526

[74] Dijkstra F, van der Sluis G, Jager-Wittenaar H et al. Facilitators and barriers to enhancing physical activity in older patients during acute hospital stay: a systematic review. Int J Behav Nutr Phys Act. 2022;19:99. 35908056 10.1186/s12966-022-01330-z PMC9338465

[75] Naughton C, de Foubert M, Cummins H et al. Implementation of a frailty care bundle (FCB) targeting mobilisation, nutrition and cognitive engagement to reduce hospital associated decline in older orthopaedic trauma patients: pretest-posttest intervention study. J Frailty Sarcopenia Falls. 2024;9:32–50. 38444547 10.22540/JFSF-09-032 PMC10910252

[76] de Foubert M, Cummins H, McCullagh R et al. Systematic review of interventions targeting fundamental care to reduce hospital-associated decline in older patients. J Adv Nurs. 2021;77:4661–78. 34240755 10.1111/jan.14954

[77] Brown CJ . After three decades of study, hospital-associated disability remains a common problem. J Am Geriatr Soc. 2020;68:465–6. 32083324 10.1111/jgs.16349

[78] Mudge AM, McRae P, Banks M et al. Effect of a ward-based program on hospital-associated complications and length of stay for older inpatients: the cluster randomized CHERISH trial. JAMA Intern Med. 2022;182:274–82. 35006265 10.1001/jamainternmed.2021.7556 PMC8749692

[79] Cohen Y, Zisberg A, Chayat Y et al. Walking for better outcomes and recovery: the effect of WALK-FOR in preventing hospital-associated functional decline among older adults. J Gerontol A. 2019;74:1664–70. 10.1093/gerona/glz025 30726886

[80] Boltz M, Resnick B, Capezuti E et al. Functional decline in hospitalized older adults: can nursing make a difference. Geriatr Nurs. 2012;33:272–9. 22401985 10.1016/j.gerinurse.2012.01.008

[81] Liu B, Moore JE, Almaawiy U et al. Outcomes of Mobilisation of Vulnerable Elders in Ontario (MOVE ON): a multisite interrupted time series evaluation of an implementation intervention to increase patient mobilisation. Age Ageing. 2018;47:112–9. 28985310 10.1093/ageing/afx128 PMC5859974

[82] Farchi T, Dopson S, Ferlie E. Do we still need professional boundaries? The multiple influences of boundaries on interprofessional collaboration. Organ Stud. 2023;44:277–98.

[83] Institute for Healthcare Improvement . Age-Friendly Health Systems. Institute for Healthcare Improvement [online]. Available from: https://www.ihi.org/Engage/Initiatives/Age-Friendly-Health-Systems/Pages/default.aspx.

[84] Mickelson Weldingh N, Kirkevold M. What older people and their relatives say is important during acute hospitalisation: a qualitative study. BMC Health Serv Res. 2022;22:578. 35488250 10.1186/s12913-022-07981-9 PMC9052562

[85] Buljac-Samardzic M, Doekhie KD, van Wijngaarden JDH. Interventions to improve team effectiveness within health care: a systematic review of the past decade. Hum Resour Health. 2020;18:2. 31915007 10.1186/s12960-019-0411-3 PMC6950792

[86] van Belle E, Giesen J, Conroy T et al. Exploring person-centred fundamental nursing care in hospital wards: a multi-site ethnography. J Clin Nurs. 2020;29:1933–44. 31408557 10.1111/jocn.15024 PMC7319433

[87] van den Berg GH, Huisman-de Waal GGJ, Vermeulen H et al. Effects of nursing nutrition interventions on outcomes in malnourished hospital inpatients and nursing home residents: a systematic review. Int J Nurs Stud. 2021;117:103888. 33647842 10.1016/j.ijnurstu.2021.103888

[88] Heinzmann J, Baumgartner C, Liechti FD. Goal-directed mobility of medical inpatients–a mini review of the literature. Front Med. 2022;9:878031. 10.3389/fmed.2022.878031 PMC9158316 35665320

[89] Cheng CM, Chiu MJ, Wang JH et al. Cognitive stimulation during hospitalization improves global cognition of older Taiwanese undergoing elective total knee and hip replacement surgery. J Adv Nurs. 2012;68:1322–9. 21988083 10.1111/j.1365-2648.2011.05842.x

[90] Jaworska N, Moss SJ, Krewulak KD et al. A scoping review of perceptions from healthcare professionals on antipsychotic prescribing practices in acute care settings. BMC Health Serv Res. 2022;22:1272. 36271347 10.1186/s12913-022-08650-7 PMC9587627

[91] Office of the Chief Nurse. Department of Health (DoH) . *Framework for Safe Nurse Staffing and Skill Mix in General and Specialist Medical and Surgical Care Settings in Ireland*. Ireland: Department of Health (Ireland), 2018.

[92] Inouye SK, Bogardus ST Jr, Baker DI et al. The hospital elder life program: a model of care to prevent cognitive and functional decline in older hospitalized patients. Hospital elder life program. J Am Geriatr Soc. 2000;48:1697–706. 11129764 10.1111/j.1532-5415.2000.tb03885.x

[93] Mudge AM, McRae P, Cruickshank M. Eat walk engage: an interdisciplinary collaborative model to improve care of hospitalized elders. Am J Med Qual. 2013;30:5–13. 24270172 10.1177/1062860613510965

[94] Bridges J, Collins P, Flatley M et al. Older people’s experiences in acute care settings: systematic review and synthesis of qualitative studies. Int J Nurs Stud. 2020;102:103469. 31862528 10.1016/j.ijnurstu.2019.103469

[95] Akunne A, Davis S, Westby M et al. The cost-effectiveness of multi-component interventions to prevent delirium in older people undergoing surgical repair of hip fracture. Eur J Orthop Surg Traumatol. 2014;24:187–95. 23412312 10.1007/s00590-013-1170-9

[96] Warren MW . Activity in advancing years. Br Med J. 1950;2:921–4. 14772519 10.1136/bmj.2.4685.921 PMC2039163

[97] Baldwin CE, Phillips AC, Edney SM et al. Recommendations for older adults’ physical activity and sedentary behaviour during hospitalisation for an acute medical illness: an international Delphi study. Int J Behav Nutr Phys Act. 2020;17:69. 32450879 10.1186/s12966-020-00970-3 PMC7249667

[98] Brown CJ, Foley KT, Lowman JD Jr et al. Comparison of posthospitalization function and community mobility in hospital mobility program and usual care patients: a randomized clinical trial. JAMA Intern Med. 2016;176:921–7. 27243899 10.1001/jamainternmed.2016.1870 PMC12983393

[99] Martínez-Velilla N, Casas-Herrero A, Zambom-Ferraresi F et al. Effect of exercise intervention on functional decline in very elderly patients during acute hospitalization: a randomized clinical trial. JAMA Intern Med. 2019;179:28–36. 30419096 10.1001/jamainternmed.2018.4869 PMC6583412

[100] Martínez-Velilla N, Abizanda P, Gómez-Pavón J et al. Effect of an exercise intervention on functional decline in very old patients during acute hospitalizations: results of a multicenter, randomized clinical trial. JAMA Intern Med. 2022;182:345–7. 35040873 10.1001/jamainternmed.2021.7654 PMC8767490

[101] Martínez-Velilla N, Sáez de Asteasu ML, Ramírez-Vélez R et al. Recovery of the decline in activities of daily living after hospitalization through an individualized exercise program: secondary analysis of a randomized clinical trial. J Gerontol A Biol Sci Med Sci. 2021;76:1519–23. 33522565 10.1093/gerona/glab032

[102] Sáez de Asteasu ML, Martínez-Velilla N, Zambom-Ferraresi F et al. Cognitive function improvements mediate exercise intervention effects on physical performance in acutely hospitalized older adults. J Am Med Dir Assoc. 2021;22:787–91. 33011095 10.1016/j.jamda.2020.08.024

[103] Martinez Velilla N, Lozano-Vicario L, Sáez de Asteasu ML et al. Could a tailored exercise intervention for hospitalised older adults have a role in the resolution of delirium? Secondary analysis of a randomised clinical trial. J Frailty Aging. 2023;12:84–5. 36629090 10.14283/jfa.2022.60

[104] Welch C, Majid Z, Greig C et al. Interventions to ameliorate reductions in muscle quantity and function in hospitalised older adults: a systematic review towards acute sarcopenia treatment. Age Ageing. 2020;50:394–404. 10.1093/ageing/afaa209 PMC7936029 33098419

[105] Sáez de Asteasu ML, Martínez-Velilla N, Zambom-Ferraresi F et al. Inter-individual variability in response to exercise intervention or usual care in hospitalized older adults. J Cachexia Sarcopenia Muscle. 2019;10:1266–75. 31407876 10.1002/jcsm.12481 PMC6903436

[106] Cuevas-Lara C, Sáez de Asteasu ML, Ramírez-Vélez R et al. Effects of game-based interventions on functional capacity in acutely hospitalised older adults: results of an open-label non-randomised clinical trial. Age Ageing. 2022;51:afab247. 10.1093/ageing/afab247 35077558

[107] NHS providers . Case Study: Introducing a Meaningful Activity Service University Hospitals of Leicester NHS Trust. NHS Providers, [Online], Available via URL: https://nhsproviders.org/media/1146/case-study-uhl-meaningful-activities-final.pdf.

[108] Breen L, Phillips SM. Skeletal muscle protein metabolism in the elderly: interventions to counteract the ‘anabolic resistance’ of ageing. Nutr Metab. 2011;8:68. 10.1186/1743-7075-8-68 PMC3201893 21975196

[109] Burton JK, Craig LE, Yong SQ et al. Non-pharmacological interventions for preventing delirium in hospitalised non-ICU patients. Cochrane Database Syst Rev. 2021;2021:CD013307. 10.1002/14651858.CD013307.pub2 PMC8407051 34280303

[110] Takeuchi I, Yoshimura Y, Shimazu S et al. Effects of branched-chain amino acids and vitamin D supplementation on physical function, muscle mass and strength, and nutritional status in sarcopenic older adults undergoing hospital-based rehabilitation: a multicenter randomized controlled trial. Geriatr Gerontol Int. 2019;19:12–7. 30358032 10.1111/ggi.13547

[111] Guo Y, Li Y, Zhang Y et al. Post-operative delirium associated with metabolic alterations following hemi-arthroplasty in older patients. Age Ageing. 2019;49:88–95. 31711096 10.1093/ageing/afz132

[112] Deer RR, Dickinson JM, Baillargeon J et al. A phase I randomized clinical trial of evidence-based, pragmatic interventions to improve functional recovery after hospitalization in geriatric patients. J Gerontol A Biol Sci Med Sci. 2019;74:1628–36. 30906944 10.1093/gerona/glz084 PMC6748704

[113] Sloan JP, Wing P, Dian L et al. A pilot study of anabolic steroids in elderly patients with hip fractures. J Am Geriatr Soc. 1992;40:1105–11. 1401694 10.1111/j.1532-5415.1992.tb01798.x

[114] Neufeld KJ, Needham DM, Oh ES et al. *Antipsychotics for the Prevention and Treatment of Delirium*. Rockville (MD): Agency for Healthcare Research and Quality (US), 2019. 31509366

[115] Shen Q-h, H-f L, X-y Z et al. Dexmedetomidine in the prevention of postoperative delirium in elderly patients following non-cardiac surgery: a systematic review and meta-analysis. Clin Exp Pharmacol Physiol. 2020;47:1333–41. 32215933 10.1111/1440-1681.13312

[116] León-Salas B, Trujillo-Martín MM, del Castillo LPM et al. Pharmacologic interventions for prevention of delirium in hospitalized older people: a meta-analysis. Arch Gerontol Geriatr. 2020;90:104171. 32682169 10.1016/j.archger.2020.104171

[117] Kehler DS, Theou O, Rockwood K. Bed rest and accelerated aging in relation to the musculoskeletal and cardiovascular systems and frailty biomarkers: a review. Exp Gerontol. 2019;124:110643. 31255732 10.1016/j.exger.2019.110643

[118] Justice JN, Nambiar AM, Tchkonia T et al. Senolytics in idiopathic pulmonary fibrosis: results from a first-in-human, open-label, pilot study. EBioMedicine. 2019;40:554–63. 30616998 10.1016/j.ebiom.2018.12.052 PMC6412088

[119] López-López L, Torres-Sánchez I, Rodríguez-Torres J et al. Does adding an integrated physical therapy and neuromuscular electrical stimulation therapy to standard rehabilitation improve functional outcome in elderly patients with pneumonia? A randomised controlled trial. Clin Rehabil. 2019;33:1757–66. 31244327 10.1177/0269215519859930

[120] Martín-Salvador A, Colodro-Amores G, Torres-Sánchez I et al. Physical therapy intervention during hospitalization in patients with acute exacerbation of chronic obstructive pulmonary disease and pneumonia: a randomized clinical trial. Med Clin. 2016;146:301–4. 10.1016/j.medcli.2015.11.009 26726117

[121] Zinglersen AH, Halsteen MB, Kjaer M et al. Can electrical stimulation enhance effects of a functional training program in hospitalized geriatric patients? Exp Gerontol. 2018;106:101–8. 29496509 10.1016/j.exger.2018.02.024

[122] Ooi H, Welch C. Obstacles to the early diagnosis and management of sarcopenia: current perspectives. Clin Interv Aging. 2024;19:323–32. 38404480 10.2147/CIA.S438144 PMC10893890

[123] MacKnight C, Rockwood K. A hierarchical assessment of balance and mobility. Age Ageing. 1995;24:126–30. 7793334 10.1093/ageing/24.2.126

[124] Martin J, Barker K. The Hierarchical Assessment of Balance and Mobility (HABAM): an underutilised tool to track physical function and estimate length of stay. Physiotherapy. 2019;105:e159.

[125] Pimentel MAF, Redfern OC, Malycha J et al. Detecting deteriorating patients in the hospital: development and validation of a novel scoring system. Am J Respir Crit Care Med. 2021;204:44–52. 33525997 10.1164/rccm.202007-2700OC PMC8437126

[126] King Z, Farrington J, Utley M et al. Machine learning for real-time aggregated prediction of hospital admission for emergency patients. NPJ Digit Med. 2022;5:104. 35882903 10.1038/s41746-022-00649-y PMC9321296

[127] Stone K, Zwiggelaar R, Jones P et al. A systematic review of the prediction of hospital length of stay: towards a unified framework. PLOS Digit Health. 2022;1:e0000017. 36812502 10.1371/journal.pdig.0000017 PMC9931263

